# Opthalmoscope in Nervous Diseases

**Published:** 1869-11

**Authors:** 


### Opthalmoscope in Nervous Diseases.

M. Bouchut, as a candidate for the next prize in Medicine and Surgery at the Academie des Sciences, has presented an additional memoir founded on his prolonged researches with the ophthalmoscope in diseases of the nervous system. He comes to the following conclusions:

"1. Diseases of the spinal cord, as acute myelitis, spinal sclerosis, locomotor ataxy, etc., frequently induce a congestive lesion of the papilla of the optic nerve, which at a later period becomes atrophic. 2. The lesions of the optic nerve produced by diseases of the cord are the result of a reflex ascending congestive action, the great sympathetic nerve acting as the inter-medium. 3. The presence of hyperæmia of the optic nerve, of reddish suffusion (*diffusion*) of the papilla, and of a total or partial atrophy of this part, coinciding with weakness and numbness of the lower extremities, indicates the existence of an acute or chronic disease of the spinal cord."—*Medical Times and Gazette.*

